# Deforestation and Benthic Indicators: How Much Vegetation Cover Is Needed to Sustain Healthy Andean Streams?

**DOI:** 10.1371/journal.pone.0105869

**Published:** 2014-08-22

**Authors:** Carlos Iñiguez–Armijos, Adrián Leiva, Hans–Georg Frede, Henrietta Hampel, Lutz Breuer

**Affiliations:** 1 Departamento de Ciencias Naturales, Universidad Técnica Particular de Loja (UTPL), Sección de Ecología, Loja, Ecuador; 2 Institute for Landscape Ecology and Resources Management (ILR), Research Centre for Bio Systems, Land Use and Nutrition (IFZ), Justus–Liebig–University Giessen, Giessen, Germany; 3 Universidad de Cuenca, Quinta Balzaín, Cuenca, Ecuador; University of Yamanashi, Japan

## Abstract

Deforestation in the tropical Andes is affecting ecological conditions of streams, and determination of how much forest should be retained is a pressing task for conservation, restoration and management strategies. We calculated and analyzed eight benthic metrics (structural, compositional and water quality indices) and a physical-chemical composite index with gradients of vegetation cover to assess the effects of deforestation on macroinvertebrate communities and water quality of 23 streams in southern Ecuadorian Andes. Using a geographical information system (GIS), we quantified vegetation cover at three spatial scales: the entire catchment, the riparian buffer of 30 m width extending the entire stream length, and the local scale defined for a stream reach of 100 m in length and similar buffer width. Macroinvertebrate and water quality metrics had the strongest relationships with vegetation cover at catchment and riparian scales, while vegetation cover did not show any association with the macroinvertebrate metrics at local scale. At catchment scale, the water quality metrics indicate that ecological condition of Andean streams is good when vegetation cover is over 70%. Further, macroinvertebrate community assemblages were more diverse and related in catchments largely covered by native vegetation (>70%). Our results suggest that retaining an important quantity of native vegetation cover within the catchments and a linkage between headwater and riparian forests help to maintain and improve stream biodiversity and water quality in Andean streams affected by deforestation. This research proposes that a strong regulation focused to the management of riparian buffers can be successful when decision making is addressed to conservation/restoration of Andean catchments.

## Introduction

Tropical deforestation is one of the most important topics worldwide associated with biodiversity loss and ecosystem degradation [1]–[3]. However, the effects of deforestation on ecological condition of tropical streams have been rarely investigated despite evidence that forest loss negatively affects these streams [4]–[7]. In Ecuador, deforestation for agriculture and/or urbanization is a critical threat for Andean ecosystems [8], reaching annual deforestation rates of 2.7% between 1989 and 2008 in the montane forests of southern Andes (Tapia Armijos, pers. communication). However many of these ecosystems provide essential environmental services including biodiversity conservation, carbon storage and water supply [9]. Several studies focused along the Ecuadorian Andean region have demonstrated that deforestation affects habitat structure and macroinvertebrate community assemblages in streams [10], litter breakdown rates [11] and hydrological processes [12]. Clearly the Ecuadorian montane streams are influenced by the surrounding landscape and several research groups have recently deployed considerable efforts to assess these relationships. Nonetheless, information gaps concerning the influence of land cover/use and spatial scale on stream condition remains, despite they affect water quality and aquatic diversity [13], [14]. These data are crucial for management and conservation of developed catchments to insure long term sustainability [15].

In other regions, organizations such as the European Environment Agency (EEA), the United States Environmental Protection Agency (EPA) and national agencies in New Zealand have identified, and are currently considering within their monitoring programs, the impacts of land cover/use changes on stream ecological conditions based on responses of macroinvertebrate communities [16]–[19]. In addition, they have developed catchment and/or riparian rehabilitation projects in order to enhance stream condition [20]. In South and Central America such extensive monitoring or restoration programs by governmental initiatives are rare, despite their potential to reduce the impact of deforestation on stream condition [13]. In this regard, a Costa Rican regulation has showed the effectiveness of protecting riparian buffers to reduce the impacts of deforestation on macroinvertebrate communities and sensitive taxa in tropical streams [4].

Several authors [10], [21]–[23] have applied a variety of macroinvertebrate metrics (e.g. Fisher's index; EPT taxa) to assess the impact of land cover/use on the ecological condition of streams in South America focusing on the structure and composition of benthic communities. These studies have confirmed changes to aquatic biodiversity and water quality because of deforestation around the streams. Nevertheless, how does vegetation cover influences stream ecological condition? In streams running through forested catchments the macroinvertebrate communities are more heterogeneous, diversity of intolerant taxa to environmental stress is higher, and abundance and variety of habitats is greater [10], [14], [21]. Likewise, streams with forested riparian areas have low variation in water temperature and less nutrients and sediment loads along the entire length [4], [13]. At the catchment scale, there is also an influence of vegetation cover on controlling hydrology and the entire diversity of the streams [12]. In this regard, estimating the extent of native vegetation cover in a catchment and the importance of riparian management and headwater forest protection to maintain and improve biodiversity and water quality of streams affected by deforestation is a pressing task for management and conservation [13], [14], [24].

Geographic Information Systems (GIS) allow researchers to explore correlations in spatial patterns with ecological features in the landscape. Allan [13] describes the ability to apply GIS tools with remote sensing data to assess the influence of land cover/use on stream ecological condition. Studies in temperate areas have described the influence of land cover and spatial scale on stream communities [25], [26] and on stream water quality [14] by applying GIS based evaluation techniques to derive land cover data and to relate them with physical, chemical and biological data.

In this study, our objective was to investigate the influence of vegetation cover at three spatial scales (local, riparian buffer and catchment) on structure and composition of macroinvertebrate communities and on water quality of headwater streams in the southern Ecuadorian Andes. These streams are located in two contrasting regions of varying intensities of anthropogenic pressure (i.e. deforestation, population growth, and farming). The questions we addressed were: what spatial scale and how much vegetation cover are associated with healthy streams based on macroinvertebrate metrics? In order to address these questions, we calculated several diversity and water quality metrics from macroinvertebrate communities, derived spatial information on land cover from GIS, and related these metrics statistically to three spatial scales and stream parameters. Finally, we addressed management and conservation problems in order to help mitigating impacts of deforestation on stream ecosystems.

## Methodology

For this study, the Ministry of Environment of Ecuador issued us a general research permit for every sampling site and to collect water samples and macroinvertebrate specimens. In the case of the Zamora upstream catchment (Zu), the Municipality of Loja granted a specific permission to work at sites Zu1–2, 4–5, 8 because these streams are used for the water supply of the city. To work at the San Francisco catchment (SF), the administration of the San Francisco Research Station and Nature and Culture International allowed us to access sites SF1–7. For all other sites we used the general research permit.

Most of macroinvertebrates collected in this study were identified at genus level and we do not know if any of these genera are endangered or protected, because of the lack of information for Ecuador.

### Study area

**Figure 1:**
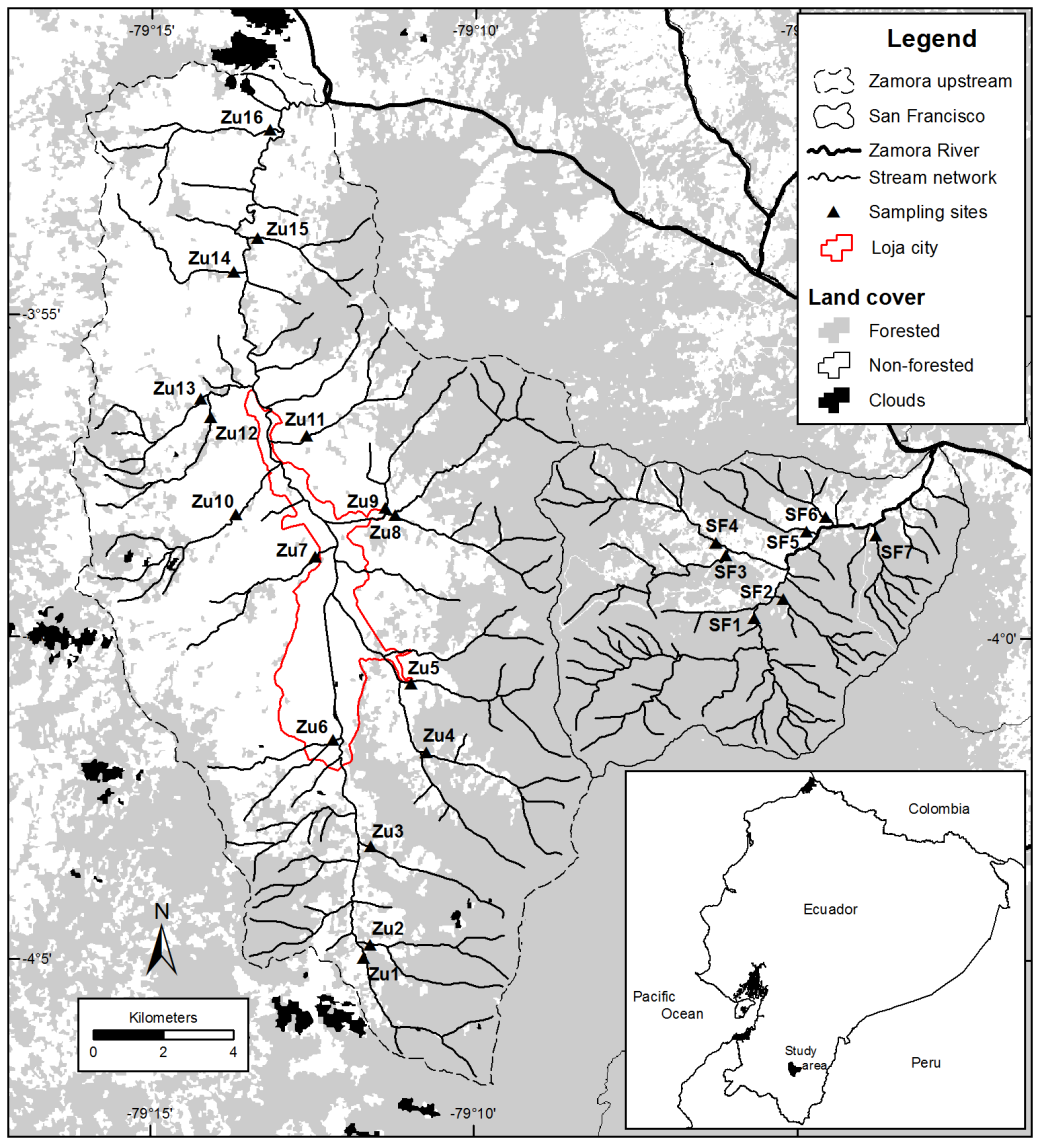
Study area and location of the sampling sites. Codes represent the sampling sites in the Zamora upstream (Zu1 to Zu16) and San Francisco (SF1 to SF7) catchments. Native vegetation cover was classified into forested and non-forested. Only the complete stream network for the study area is shown. Catchment characteristics are further shown in Table S1.

We studied 23 streams of two headwater catchments of the Zamora River basin, located in the mountain forests in Ecuadorian southern Andes (Figure 1). The Zamora upstream catchment covers an area of 276.2 km^2^ and ranges between 2,020 to 3,250 m a.s.l. It is the most populated catchment within the Zamora River basin with approximately 120, 000 inhabitants in the city of Loja. The San Francisco catchment is located in a sparsely populated area of the Zamora River basin with an area of 75.3 km^2^ and an elevation range from 1,800 to 3,250 m a.s.l. The natural vegetation in both study catchments is evergreen mountain rain forests, humid shrub, cloud forest and páramo [27], [28]. Anthropic activities in both catchments have substituted the original vegetation mainly to pasture, and to a lesser extent to cropland, pine (*Pinus*) and eucalyptus (*Eucalyptus*) plantations [29]. Urban development has only taken place in the Zamora upstream catchment and has spread along its main river. Both catchments have similar climatic conditions with a wet season from May to September and a dry season from October to April. The average annual precipitation and temperature in the Zamora upstream and San Francisco catchments are about 2,200 mm and 15.2°C respectively.

### Stream measurements and macroinvertebrates sampling

We sampled 16 streams in the Zamora upstream catchment and 7 streams in the San Francisco catchment. Sampling focused on riffles with an altitudinal variation of 350 m among sites. Every site consisted of a 30–60 m reach placed at the outlet of each stream in order to capture the influences of land use upstream on macroinvertebrate communities. According to the sampling frequency applied in other studies with benthic macroinvertebrates both in Ecuador and in other Neotropical regions [4], [10], [11], [30]–[34], we collected physical–chemical and macroinvertebrate data during both dry (November 2010; December 2012) and wet seasons (May 2011; April 2012) at the same stream reach. Physical–chemical parameters such as pH, temperature (*T*), specific conductance (SC), dissolved oxygen (DO), were assessed in–stream in each of the sampling periods (Table S1) with a portable YSI 556 Multi Probe System and were averaged from several measurements during the sampling campaigns. Grab samples were taken for measuring further water chemistry at the laboratory of the Universidad Técnica Particular de Loja (UTPL) using standard methods [35]: nitrate (NO_3_^−^), phosphate (PO_4_^3−^), biochemical oxygen demand during decomposition in a period of 5 days (BOD_5_), total coliforms (TC) and turbidity (Table S1).

For sampling of macroinvertebrate communities several methods exist and each one provides different results, nevertheless a combination of sampling methods is preferable [30]. In this study, qualitative macroinvertebrates samples were taken along the stream reach applying two sampling methods: 1) collecting macroinvertebrates directly in their stream habitats from stones and leaves, and 2) using a D–frame net (500 µm mesh) for kick sampling at 3–6 points selected randomly. Collection method 1 was applied during 10 min along the reach to remove all visible macroinvertebrates from stones and collect leaves respectively. Selected stones collected were totally submerged and had an average diameter of 20 cm approximately. Leaves were stored in a plastic bag and rinsed with tap water to remove macroinvertebrates in the laboratory. For collection method 2, macroinvertebrates were sampled by kicking the substrate while holding the D–frame net downstream at each point. The application of this technique was based on its suitability for sampling in a variety of stream substrates and depths [36], [37]. Both collections were combined into a single collection per site in order to avoid differences on macroinvertebrate communities due to seasonality of the Ecuadorian Andes [38], [39]. All macroinvertebrates were preserved in 95% ethanol and brought to the laboratory to be sorted and identified to the lowest taxonomical level possible using identification keys for South American macroinvertebrates [40], [41].

### GIS data and vegetation cover

The Geographic Information System software ArcGIS 9.3 was used to evaluate spatial digital information. Catchments were delineated from a Digital Elevation Model (DEM) with a10 m resolution, available at the Research Unit 816 (RU 816) database (www.tropicalmountainforest.org/) of the Deutsche Forschungsgemeinschaft (DFG). Stream networks were obtained from the national cartography created by the National Mapping Agency (IGM; Instituto Geográfico Militar).

Based on Allan [13], the influence of vegetation cover (VC) on stream condition was evaluated at three spatial scales: 1) catchment scale representing the entire area upstream of the sampling site; 2) riparian scale, using a buffer of 30 m on each bank along the entire length of the stream; and 3) local scale, comprised by the same buffer width along a 100 m reach upstream of the sampling site. Buffer width chosen was based on regulations by local governments (called Municipal Ordinances) in Ecuador. These ordinances reflect several law articles of the Ecuadorian Government in terms of water resources protection, but it does not specify or recommend an amount of riparian vegetation.

A 2010 land use map provided by M.F. Tapia–Armijos (pers. communication) was used to quantify VC within each catchment. This map was generated for southern Ecuador from ASTER images (http://asterweb.jpl.nasa.gov/) with a minimum mapping unit of 1 ha and classified into eight main land use types: páramo, forest, shrub, pasture, crop, plantation, urban and un–vegetated. In Table 1 we show the land use and percentage area covered by each land use type within the 23 studied catchments, with forest and pasture classes as the most dominating ones. Urban land use was absent or very poorly represented. For this study we aggregated this classification to two land cover categories, i.e. forested and non–forested. Forest class included all types of native vegetation and non–forest class all types of disturbed areas (pasture, cropland, plantation, urban, un–vegetated). The forest class was used to determine the VC only at catchment scale, because resolution and accuracy may limit the interpretation of riparian land cover in mountainous areas [13], [25], [42]. In order to address the riparian and local scales, the VC within the 30 m buffer was digitized and interpreted from 2011 rectified colored orthophotographs (0.30 m resolution). These orthophotographs were obtained and provided by the Ecuadorian project SIGTIERRAS (Ministerio de Agricultura, Ganadería, Acuacultura y Pesca; Proyecto Sistema Nacional de Información y Gestión de Tierras Rurales e Infraestructura Tecnológica).

**Table 1:** Land use characteristics and area (%) of each land use type of the 23 studied catchments.

Catchment	Páramo	Forest	Shrub	Pasture	Plantation	Crop	Urban	Un-vegetated
Zu1	0.8	69.4	13.4	13.6	0.3	2.3	0.0	0.2
Zu2	9.3	61.1	14.4	12.5	0.5	2.1	0.0	0.0
Zu3	1.4	59.4	10.7	25.0	0.7	2.6	0.0	0.1
Zu4	2.8	46.9	19.3	25.2	0.5	5.3	0.0	0.2
Zu5	4.4	54.9	27.9	10.4	0.9	1.4	0.1	0.1
Zu6	0.3	8.8	17.6	66.0	2.2	2.9	1.5	0.7
Zu7	0.0	4.6	13.2	71.1	0.8	9.0	0.0	1.2
Zu8	2.7	39.0	25.6	30.9	0.7	1.0	0.0	0.2
Zu9	0.5	41.0	12.4	35.9	1.5	8.3	0.2	0.1
Zu10	0.4	8.0	28.0	47.1	2.5	13.4	0.0	0.6
Zu11	0.4	33.7	9.1	42.0	2.7	11.9	0.0	0.2
Zu12	0.2	17.6	4.7	57.3	2.9	16.3	0.0	0.9
Zu13	0.4	16.0	19.2	48.9	1.2	13.5	0.0	0.7
Zu14	0.0	6.5	0.1	73.2	1.9	15.0	0.0	3.3
Zu15	0.0	51.9	9.9	32.9	1.1	4.0	0.0	0.2
Zu16	0.0	15.6	0.2	72.4	2.5	7.0	0.0	2.3
SF1	0.0	82.3	5.3	5.6	0.0	0.0	0.0	6.8
SF2	2.0	93.5	4.0	0.0	0.0	0.0	0.0	0.5
SF3	0.0	78.0	5.0	15.8	0.3	0.0	0.0	0.9
SF4	1.9	87.6	3.7	6.3	0.1	0.0	0.0	0.3
SF5	0.4	81.8	3.0	14.1	0.4	0.0	0.0	0.3
SF6	0.4	76.3	2.8	18.9	1.2	0.0	0.0	0.4
SF7	2.0	92.0	3.9	2.0	0.0	0.0	0.0	0.1

### Data analysis

We calculated nine indices (also referred as metrics) to describe ecological conditions of the streams. We calculated three structural, two compositional and four water quality indices (Table 2) that allowed us assessing stream conditions in response to increasing VC. The structural indices were richness, Fisher's index, and evenness (Pielou's index). For compositional indices we classified macroinvertebrates by functional feeding groups (FFG) according to [43], [44], but we only used percentage of scrappers and shredders because they have an expected decreasing response to perturbation [36], in our case deforestation.

**Table 2:** Results of regression analysis between calculated macroinvertebrate metrics and percentage of vegetation cover at multiple spatial scales.

Spatial scale	Catchment		Buffer		Local	
Metric	*R^2^*	*p*	*R^2^*	*p*	*R^2^*	*p*
Structural						
Richness	0.30	<0.01	0.34	<0.001	0.10	0.13
Fisher's index	0.50	<0.001	0.38	<0.001	0.01	0.30
Evenness	0.24	<0.01	0.11	0.11	0.00	0.93
Compositional						
% Scrappers	0.02	0.57	0.01	0.72	0.02	0.55
% Shredders	0.42	<0.001	0.46	<0.001	0.12	0.09
Water Quality						
WQI†	0.56	<0.001	0.50	<0.001	0.12	0.11
EPT index	0.53	<0.001	0.59	<0.001	0.03	0.39
BMWP/Col	0.44	<0.001	0.46	<0.001	0.17	0.05
%5 Dominant taxa	0.85	<0.001	0.60	<0.001	0.02	0.50

† WQI is the unique metric derived from water chemistry data showed in Table S1.

Water quality was assessed with the Water Quality Index (WQI), the EPT index, the Biological Monitoring Working Party index adapted to Colombia (BMWP/Col), and the %5 Dominant taxa. WQI is a single score composed of several measurements of various physical, chemical and bacteriological parameters, where low WQI values show high influence of environmental stress [45]. EPT index shows the taxa richness within the macroinvertebrate groups which are considered sensitive to stressors, and therefore should increase with increasing water quality [46]. BMWP/Col index provides a single value at the family level, representative of the organism's tolerance to stressors, where high tolerance to pollution corresponds to low BMWP scores [40]. %5 Dominant taxa is a measure of the abundance of the five most dominant taxa to the total number of organisms in the sample at each site [36]. When a community is dominated by few taxa it will have high dominance values, thus indicating the community is under the influence of environmental stress.

Two statistical approaches have been used to evaluate relationships between vegetation cover/land use and ecological indices; these are regression [14], [42], [47] and ordination analyses [4], [10], [48]. Prior to analyses, macroinvertebrate abundance data (*x*) were transformed by log_10_ (*x*) to reduce the effect of large numbers in single taxa (e.g. *Baetodes*). Rare taxa, i.e. taxa represented by three or less individuals in a sampling site, were excluded in order to avoid the influence of these taxa in the ordination analysis [49]. Because explanatory variables did not have a uniform scale, stream parameters were log_10_ (*x*+1) transformed in order to avoid the problem with 0 values and reduce the range of data. All of these variables were analyzed by pairs in scatterplot matrices, and through an exploratory Redundancy Analysis (RDA) to identify the least correlated and most informative variables (PO_4_^3−^, DO, TC and turbidity).

An important issue was to identify whether ecological condition of the streams was attributable to a land use type and/or a stream parameter. We used a stepwise multiple linear regression (SMLR) with calculated macroinvertebrate metrics as dependent variables against land use and PO_4_^3−^, DO, TC and turbidity to determine which of those independent variables affect our response variables. SMLR was applied with the ‘stepAIC’ function of ‘MASS’ package in R [50] using backward elimination of variables and Akaike Information Criterion for variables or predictors selection. In addition, we estimated the relative importance of predictors based on their contribution to *R^2^* using boostrap (‘relaimpo’ package). All *p*–values were adjusted with a Bonferroni correction for multiple comparisons during regression analyses. These analyses indicated that forest class (i.e. VC) was consistently more informative to explain macroinvertebrate metrics than other land use types or stream parameter (see Table S2 for SMLR results). Therefore, to examine the linear dependency of each of our response variables with increasing VC, macroinvertebrate metrics (dependent) were regressed against percentages of VC at each spatial scale (independent) using the method of least mean squares (‘lm’ function of ‘stats’ package).

We used a RDA (‘rda’ function of ‘vegan’ package) to identify macroinvertebrate community assemblages associated with percentages of VC, PO_4_^3−^, DO, TC and turbidity. RDA evaluates multivariate response data and is a powerful tool of constrained ordination in community ecology [51]. RDA works on a matrix *Y* of response data (e.g. macroinvertebrate data) and a matrix *X* of explanatory variables (e.g. water chemistry, VC). It identifies groups and shows the vector fitting of variables used in the analysis. While the vector fitting is commonly used for environmental interpretation in ordination diagrams, this implies a linear relationship between ordination scores and the environment variables and may not always be appropriate if it is desired to understand the ordination of studied sites or species in relation to one or more variables. Thus, we fitted the surface of the best predictor variable, identified with an ANOVA of the RDA results, to detect how macroinvertebrate communities are grouped and spread in relation to this variable. We examined whether macroinvertebrate communities were different between those groups using an Analysis of Similarities (ANOSIM; ‘anosim’ function of ‘vegan’ package). ANOSIM is a nonparametric procedure that provides a test of whether there is a significant difference between two or more groups of sampling units.

## Results

### Stream condition and vegetation cover

Relationships between VC and all metrics are generally stronger at catchment and buffer scale than at local scale where associations are not apparent (Table 2). Significant correlations are found on catchment and buffer scale in 90% and 80% of all cases. Within the structural metrics, only evenness does not show significant relationship with the percentage of VC at buffer scale (*R^2^* = 0.11; *p* = 0.11). In general, correlations decrease with smaller spatial scales, disappearing completely at local scale.

As for compositional metrics, only one FFG shows a strong relationship with VC at the two larger scales (*p*<0.001). The percentage of shredders is more related to VC at catchment and buffer scales (Table 2). Scrappers show weaker regressions at all spatial scales (*R^2^*<0.02). At local scale no significant correlation existed at all.

**Figure 2:**
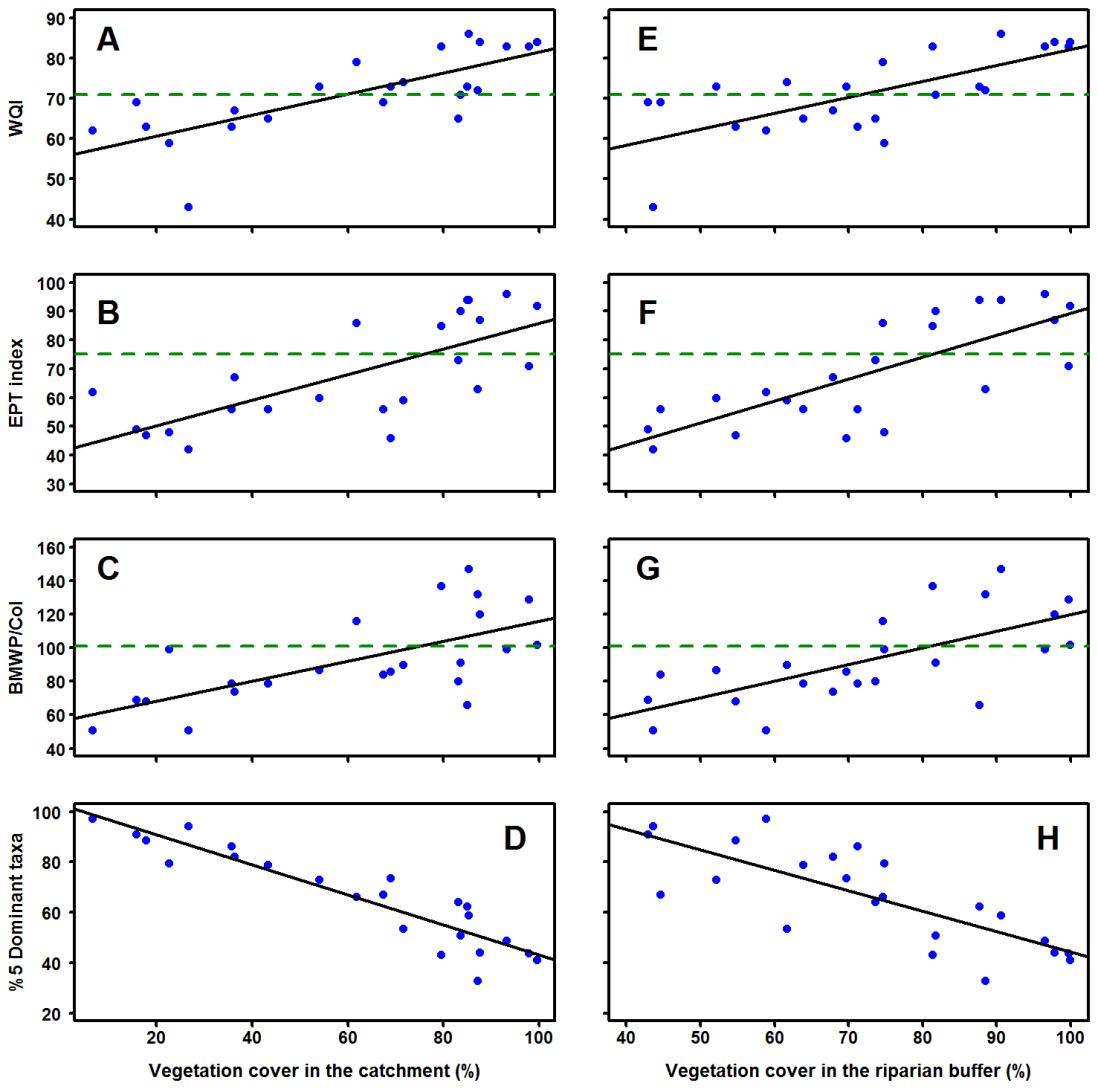
Water quality indices plotted against vegetation cover (%) at catchment and buffer scales. Horizontal green dashed lines represent thresholds of good water quality defined for each metric in the literature. Only the %5 Dominant index does not have ranges of tolerance, but high percentages indicate stress in the communities. For both spatial scales *R^2^* values are over 0.44 with a *p*–value <0.001, see Table 2 for specific values of each regression.

All water quality metrics have stronger relationships at catchment and buffer scale than at local scale (*R^2^*>0.44 and *p*<0.001, Table 2). Within water quality metrics, the %5 Dominant taxa depicts the highest correlation across all scales with 0.85 on catchment scale. Regression slopes indicate significant differences in water quality when VC increases at both spatial scales. However, there are some streams with a large area covered by forest at catchment and buffer scale which present low values in water quality indices (Figure 2B–C; F–G). For example, in both spatial scales the EPT index increase with VC (*R^2^* = 0.53; *p*<0.001 and *R^2^* = 0.59; *p*<0.001), but there is one site with approximately 70% of forest cover whose index value is less than 50, indicating fairly poor water quality. In addition, the strongest relationship is inversely correlated between the %5 Dominant taxa and the percentage of VC in the catchment (*R^2^* = 0.85; *p* = 0.001). As a general pattern most of the macroinvertebrate metrics indicate good water quality when the percentage of native VC is higher than 70% within the catchment and the riparian buffer (see Figure 2).

### Macroinvertebrate communities and vegetation cover

**Figure 3:**
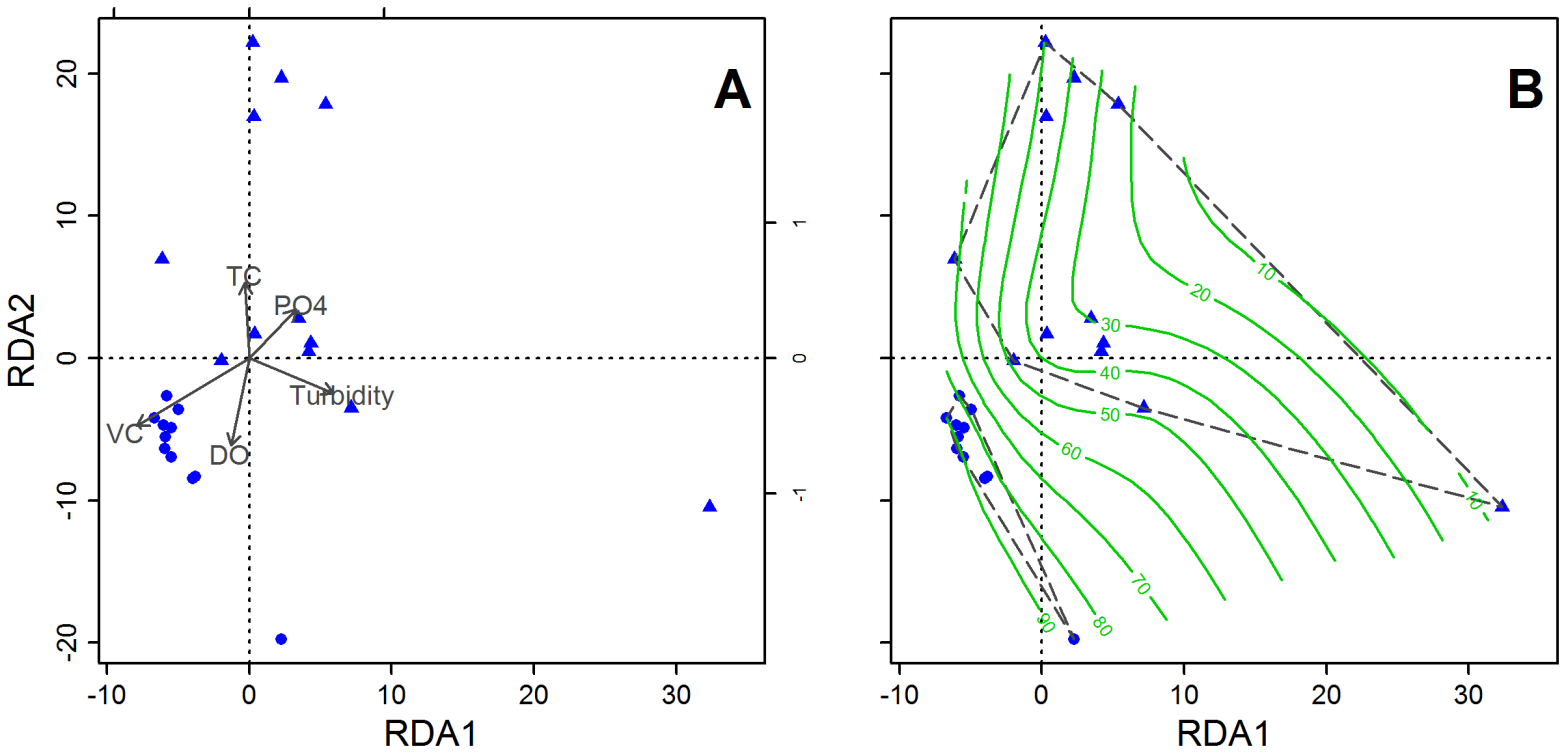
Redundancy Analysis (RDA) of macroinvertebrate community assemblages from catchments with different vegetation covers (VC). This ordination analysis detected two groups of macroinvertebrate assemblages: solid circles represent macroinvertebrate communities in catchments with 70–95% in VC and triangles indicate macroinvertebrate communities in catchments with less than 70% in VC. Each symbol represents the community of a single catchment, i.e. macroinvertebrate community at catchment SF3. A Shows the RDA ordination with vector fitting of five variables selected during analysis. DO =  dissolved oxygen, TC =  total coliforms, PO_4_ =  phosphate. B Displays in green curves the fitted surface only for the percentage of VC, which is the most explicative variable of the ordination (65%). Dashed lines enclose and separate the detected groups.

All structural indices are related with VC at catchment scale rather than at other spatial scales (Table 2). Thus, we decided to further analyze the community assemblage patterns related to catchment forest cover. A correlation analysis run for those variables showed scatterplot matrices and correlation values from 0 to 1.We defined a decision threshold of 0.50 to remove those variables which are correlated with more than one variable. Remaining variables include VC, DO, PO_4_^3−^, TC and turbidity. RDA reveal that 51% of the variation in the data can be explained by these five selected variables and the ANOVA identified that catchment VC is the best predictor for macroinvertebrate composition in the ordination (*p*<0.001). In Figure 3A, most variables are spread along negative axis 1 and negative axis 2, with VC, PO_4_^3−^ and turbidity being the most influential factors for axis 1, while DO and TC are more associated with axis 2. Figure 3B depicts the fitted surface of the best predictor variable (i.e. VC) to detect how macroinvertebrate communities are grouped and spread in relation to VC. Thus the ordination plot illustrates two visibly distinguishable groups; one group associated to catchments with a forest cover above 70% which is even more aggregated than the other group related to lower VC. The ANOSIM indicates that both groups of macroinvertebrate assemblages were strongly dissimilar between both ranges of VC (*R* = 0.55; *p* = 0.001). Both RDA plot and Bray–Curtis distance of the ANOSIM suggest that macroinvertebrate assemblages differ in taxonomical composition when there is a decline in the percentage of VC within the catchments.

Overall, macroinvertebrate communities in the 23 study streams present specific patterns. They are composed of a total of 13,512 individuals belonging to 11 orders, 33 families and 53 genera (data available at PANGAEA – Data Publisher for Earth & Environmental Science; doi:10.1594/PANGAEA.831687). Only two taxa (taxa–1 =  Empididae; taxa–2 =  Pyralidae) were not identified to genera level.

Comparing occurrence and abundance patterns between both groups of catchments (i.e. less than 70% and greater than 70% VC) we detected several singularities. In catchments with a VC between 70 to 100% we collected 4,061 individuals of 11 orders. In contrast 9,451 individuals of nine orders were found in catchments with less VC (Basommatophora and Lepidoptera were absent). We found 33 families, all of them present in catchments highly forested; whilst 10 families were absent in catchments with less than 70% forest cover (Calamoceratidae, Ceratopogonidae, Coenagrionidae, Dolichopodidae, Gyrinidae, Odontoceridae, Physidae, Psephenidae, Ptilodactylidae, and Pyralidae). With regard to the 53 genera found, only *Scirtes* (Coleptera) and *Molophilus* (Diptera) were absent in catchments above 70% forest cover, and 25 genera were absent in catchments with lower forest cover showing the greatest difference between both groups of catchments at this taxonomic level. Here genera absent at <70% catchment VC were: *Physa* (Basommatophora); *Noelmis*, *Phanacerus*, *Dineutus*, *Anchytarsus*, *Psephenops*, *Elodes* (Coleoptera); *Probezzia*, *Aphrosytus*, *Hexatoma* (Diptera); *Andesiops*, *Moribaetis*, *Haplohyphes*, *Tricorythodes*, *Farrodes* (Ephemeroptera); taxa–2 (Lepidoptera); *Argia* (Odonata); *Phylloicus*, *Leptonema*, *Macrostemum*, *Grumichella*, *Nectopsyche*, *Triplectes*, *Marilia*, *Cernotina* (Tricoptera).

A trend can be observed here as well, confirmed by the ANOSIM, as there are differences in macroinvertebrate compositions between both groups of catchments (Table 3). We detect an increase for almost all metrics of the macroinvertebrate communities with an increase of the percentage of VC in the catchment, except for evenness where no changes are observed.

**Table 3:** Diversity patterns of macroinvertebrate communities in both groups of catchments identified in RDA ordination plot (Figure 3B). VC =  catchment vegetation cover.

Vegetation cover	<70% VC		>70% VC		Response to increasing VC
Metric	Mean	SD	Mean	SD	
Structural					
Richness	14.3	2.1	20.1	6.2	Increase
Fisher's index	2.5	0.4	4.9	1.3	Increase
Evenness	0.7	0.1	0.7	0.1	Variable
Compositional					
% Scrappers	26.0	16.3	30.6	17.3	Increase
% Shredders	0.3	0.8	3.1	2.6	Increase

## Discussion

### Stream condition and vegetation cover

Our results show a stronger relationship between VC and the macroinvertebrate metrics at catchment and buffer scales. This suggests an important linkage between headwater and riparian forests to conserve diversity and water quality of the streams in Ecuadorian Andean catchments. We observe that stream condition begins to improve when native VC within the catchment is over 70% (see Figure 2). Therefore our suggestion is to retain at least 70% native VC within the catchment in order to keep sufficient ecological condition of the streams. Such a percentage of VC can contribute significantly to riparian management for protection or restoration of watersheds where forest has been replaced by grasslands. Further, our findings show that local scale strategies (e.g. conserving small forest patches in riparian areas) do not improve water quality and are unlikely to improve the overall ecological condition of streams. According to [13], [47], the maintenance of ecological conditions may require conservation of more than only a few native forest patches, and efforts should focus on considering larger spatial patterns that have more important consequences for stream conservation.

Macroinvertebrates diversity is more related to native VC at catchment and buffer scale than at a local scale. Many studies have documented the influence of upstream vegetation on stream ecological condition downstream, especially on water chemistry and habitat conditions [13], [14], [52]. For example, DO increases as the stream enters the forest [53]. Likewise, the similar relationships shown between water quality metrics and VC at catchment and buffer scale is not surprising given the strong longitudinal connectivity in a riverine landscape [13], [54]. Riparian forests can influence water chemistry and habitat quality, which are directly linked to the macroinvertebrate metrics and help to minimize the effects of catchment deforestation on streams [4], [42], [55].

In this study, there were two particular sampling points in streams of catchments Zu4 and Zu8 (see Figure 1 and Table S1). Both had low values in water quality indices but a relatively high VC (60–68%). We assume that livestock and minor roads which are located parallel to the streams influence these metrics. For example, in [4] the authors found a stream affected by inputs of sediments and nutrients derived from roads and livestock, despite having a wide riparian buffer. We believe those elements are affecting the macroinvertebrate composition of very well forested catchments as discussed below.

### Macroinvertebrate communities, indicators and vegetation cover

Analyzing the composition of macroinvertebrate communities of 23 Andean streams, we are able to identify direct relationships with the coverage of the catchment's native forest. The difference between macroinvertebrate assemblages increases when decreasing the percentage of native VC. We find a group with a more aggregated distribution when VC is large in the RDA plot. This pattern was also observed by [14] in a larger sample of streams in New Zealand. It supports the premise that deforestation is affecting the macroinvertebrate community assemblages in Ecuadorian Andean streams as in other Neotropical streams [4], [21], [56].

The difference between both groups of macroinvertebrate communities detected in the RDA is also revealed in the ANOSIM. Bücker et al. [10] found similar patterns in the San Francisco catchment having less variation at structural and compositional level and higher water quality metric values in forested than in deforested streams. Comparable results were also found in other Andean streams [11] and Amazon streams [21] of northern Ecuador. Nonetheless, among all metrics used here and mostly applied in similar studies, %5 Dominant taxa reveals a superior fitting to the regression slope despite it has not been used to reflect deforestation effects on streams before. In [36], authors denoted that high values of this metric indicate a community under environmental stress.

In terms of the FFG composition, we observe a direct response to increasing VC in both groups of macroinvertebrates (Table 3). In average, the abundance of shredders present in streams with >70% VC in catchment is always higher than in less forested catchments. Shredders abundance is typically linked to leaf litter from riparian vegetation and it is sensitive to riparian zone impacts [43], [57], [58]. Shedders could be considered as an indicator of deforestation in Andean streams, despite proportion of shredders in macroinvertebrate communities in our catchments are always small (see Table 3). According to [4], [5] this seems to be a general pattern in tropical streams. On average, scrapers abundance is slightly higher in more forested catchments in contrast to other Neotropical streams where their abundance is higher in less forested streams [4]. Additionally no association between scrappers and VC was detected.

### Management and conservation

Allan et al. [13], have recommended that it is preferable to retain as much forest cover as possible in a catchment to preserve natural stream condition. However, in tropical regions like Ecuadorian Andes, deforestation has been increasing throughout the years affecting a large part of catchments along the Andes [29], [59], [60]. Deforestation has stopped in the upper Andean catchments because of topography and accessibility, preserving headwater forests and/or páramo [29]. However, this native vegetation in the headwaters is not enough to sustain healthy streams. In Andean countries, human activities are especially concentrated on midstream sections and valleys of catchments affecting water/habitat quality downstream and other rivers after the confluence. For example, within Guayas river basin in Ecuador there are several cities and in combination with intensive agricultural land use they are affecting the entire river network and its water quality [61]. Our results suggest that retaining over 70% native forest cover in Andean catchments now dominated by grasslands and keeping the riparian buffers, the water quality and stream biodiversity could be enhanced or at least preserved. However, this task is not so easy to achieve if there is not a strong regulation at national level.

The Ecuadorian constitution defines the right to water and promotes the protection, restoration and management of basins and water resources; but it does not mention how much forest needs to be retained in a catchment or a recommended width for riparian buffers. Best conservation actions that intend maintaining as much forest as possible have been focused on catchments for drinking water supply from initiatives of local governments (Municipalities) supported by non–governmental organizations, through the establishment of trust funds for catchment conservation. Also, local governments through Municipal Ordinances have decided to protect riparian strips with different buffer widths (15–30 m), depending if the stream is running in the city or outside the urban boundary. Riparian buffers could considerably reduce the effects of deforestation on benthic communities and water quality, but there is need for a proper management of riparian areas [4]. Unfortunately, Municipal Ordinances in Ecuador do not include the management of riparian buffers. Here, we assessed the effect of a 30 m buffer width on stream condition showing strong associations with the water quality metrics. Therefore, we also recommend protection and an effective management of native riparian forest buffers in Andean streams to assist headwater forests to sustain ecological condition of the streams.

We did not evaluate any management approaches of riparian areas, but we would like to recommend some strategies that governments can include in national regulations. We believe that the most important problems that managers will face in Andean streams are livestock density, residential areas and parallel roads along riparian zones which affect water quality and macroinvertebrate communities in forested catchments. Therefore, these problems must be addressed; for example, livestock exclusion jointly with a long–term research program to assess its effectiveness [62]; or an in–depth monitoring to further study road effects on stream and riparian vegetation [63]. Another important issue to be considered is the restoration of riparian vegetation, mainly because it enhances water quality, as our study indicates. Restoration positively affects habitat and macroinvertebrate community heterogeneity [48] and can be ecologically successful by using native plant species and avoiding the use of invasive plant species, which can affect structure and functionally of streams [64]. Finally, as mentioned by [4] we would like to emphasize that “riparian forest buffers are not a replacement for continuous forest, but can promote biodiversity and stream ecosystem integrity in tropical catchments affected by deforestation”.

## Supporting Information

Table S1 **List of environmental variables considered in the 23 studied Andean streams.** Data of water parameters are means obtained from several measurements in two sampling campaigns. Altitude was determined once at the sampling sites. Catchment area and percentage of vegetation cover (VC) at three spatial scales were calculated in ArcGIS 9.3 Geographic Information System software. T =  Temperature, SC =  Specific Conductance, DO =  Dissolved Oxygen, NO_3_^−^ =  Nitrate, PO_4_^3−^ =  Phosphate, BOD_5_ =  Biochemical Oxygen Demand in 5 days. Turbidity in Nephelometric Turbidity Units or NTU and Total Coliforms (TC) as Colony Forming Units or CFU.
(DOCX)

Table S2 **Stepwise multiple linear regression analysis between macroinvertebrate metrics, land use and stream parameters for 23 Andean streams.** Degrees of freedom for all regressions were 1, 20. AIC =  Akaike Information Criterion used for selection of predictor variables. DO =  Dissolved Oxygen, PO_4_^3−^ =  Phosphate, TC =  Total Coliforms.
(DOCX)
