# Supplementary material for: Deforestation and Benthic Indicators: How Much Vegetation Cover Is Needed to Sustain Healthy Andean Streams?
Source: PLoS One. 2014 Aug 22;9(8):e105869. doi: 10.1371/journal.pone.0105869 (PMC4141824; doi:10.1371/journal.pone.0105869)
Supplement: Table S1 — List of environmental variables considered in the 23 studied Andean streams. Data of water parameters are means obtained from several measurements in two sampling campaigns. Altitude was determined once at the sampling sites. Catchment area and percentage of vegetation cover (VC) at three spatial scales were calculated in ArcGIS 9.3 Geographic Information System software. T = Temperature, SC = Specific Conductance, DO = Dissolved Oxygen, NO3 − = Nitrate, PO4 3− = Phosphate, BOD5 = Biochemical Oxygen Demand in 5 days. Turbidity in Nephelometric Turbidity Units or NTU and Total Coliforms (TC) as Colony Forming Units or CFU. (DOCX) [file pone.0105869.s001.docx]

Table S1. List of environmental variables considered in the 23 studied Andean streams. Data of water parameters are means obtained from several measurements in two sampling campaigns. Altitude was determined once at the sampling sites. Catchment area and percentage of vegetation cover (VC) at three spatial scales were calculated in ArcGIS 9.3 Geographic Information System software. T= Temperature, SC= Specific Conductance, DO= Dissolved Oxygen, NO_3_^¯^= Nitrate, PO_4_^3¯^= Phosphate, BOD_5_= Biochemical Oxygen Demand in 5 days. Turbidity in Nephelometric Turbidity Units or NTU and Total Coliforms (TC) as Colony Forming Units or CFU.

| Catchment / Stream | Code | Area (ha) | Catchment VC (%) | Buffer VC (%) | Local VC (%) | Altitude (m) | Channel width (m) | Channel depth (cm) | pH (-) | T (°C) | SC (µS/cm) | DO (mg/L) | NO_3_^¯^ (mg N/L) | PO_4_^3¯^ (mg P/L) | BOD_5_ (mg O_2_/L) | Turbidity (NTU) | TC (CFU/100 ml) |
| --- | --- | --- | --- | --- | --- | --- | --- | --- | --- | --- | --- | --- | --- | --- | --- | --- | --- |
| Curitroje | Zu1 | 776.2 | 83.6 | 81.7 | 0.0 | 2,254 | 3.1 | 15.0 | 6.5 | 17.9 | 39.4 | 7.1 | 1.6 | 0.24 | 5.0 | 3.0 | 800 |
| Mónica | Zu2 | 927.6 | 84.9 | 87.6 | 6.8 | 2,243 | 3.0 | 13.5 | 6.4 | 16.4 | 24.4 | 7.6 | 0.9 | 0.07 | 5.0 | 1.9 | 740 |
| La Amanda | Zu3 | 740.8 | 71.6 | 61.6 | 0.0 | 2,246 | 3.2 | 11.5 | 7.7 | 17.5 | 49.8 | 7.8 | 2.5 | 0.09 | 2.3 | 9.7 | 860 |
| El Carmen | Zu4 | 1,124.8 | 68.9 | 69.7 | 0.0 | 2,235 | 2.7 | 10.0 | 7.1 | 16.2 | 36.6 | 7.9 | 1.2 | 0.33 | 3.7 | 4.0 | 1,200 |
| Mendieta | Zu5 | 922.3 | 87.1 | 88.4 | 31.8 | 2,154 | 2.4 | 14.8 | 7.4 | 15.6 | 48.9 | 7.8 | 1.9 | 0.09 | 7.3 | 5.0 | 1,000 |
| de Alumbre | Zu6 | 678.3 | 26.7 | 43.6 | 0.0 | 2,139 | 2.1 | 12.3 | 6.3 | 16.2 | 120.6 | 6.3 | 2.7 | 0.41 | 9.3 | 130.6 | 8,500 |
| Las Pavas | Zu7 | 762.4 | 17.8 | 54.7 | 62.3 | 2,095 | 2.4 | 11.6 | 6.8 | 18.6 | 63.5 | 7.1 | 2.1 | 0.33 | 5.0 | 63.2 | 6,800 |
| de Sangre | Zu8 | 1,147.3 | 67.3 | 44.6 | 0.0 | 2,092 | 3.0 | 11.8 | 6.6 | 18.2 | 30.5 | 7.2 | 2.2 | 0.23 | 5.0 | 12.1 | 8,000 |
| El Salado | Zu9 | 639.2 | 54.0 | 52.1 | 0.0 | 2,099 | 2.6 | 11.0 | 7.2 | 20.8 | 54.4 | 7.1 | 2.0 | 0.19 | 2.2 | 11.2 | 1,300 |
| La Banda | Zu10 | 1,035.1 | 36.3 | 67.9 | 11.6 | 2,085 | 3.1 | 14.2 | 6.4 | 17.2 | 88.3 | 7.2 | 2.1 | 0.08 | 5.0 | 13.4 | 5,300 |
| Paccha | Zu11 | 712.6 | 43.2 | 63.8 | 16.4 | 2,072 | 2.0 | 16.7 | 7.1 | 18.5 | 51.3 | 7.8 | 1.9 | 0.11 | 2.3 | 3.0 | 1,300 |
| Carigán | Zu12 | 586.7 | 22.6 | 74.8 | 86.3 | 2,125 | 2.3 | 15.6 | 7.2 | 18.6 | 95.6 | 7.2 | 1.6 | 0.18 | 3.7 | 22.1 | 1,100 |
| Tenería | Zu13 | 619.6 | 35.6 | 71.2 | 56.9 | 2,098 | 2.9 | 14.3 | 7.6 | 18.6 | 39.4 | 7.5 | 1.2 | 0.09 | 2.3 | 5.0 | 800 |
| Chirimoyo | Zu14 | 702.6 | 6.6 | 58.8 | 18.4 | 2,060 | 2.6 | 15.1 | 7.8 | 19.3 | 188.7 | 7.4 | 1.0 | 0.30 | 5.0 | 199.5 | 140 |
| Sevilla | Zu15 | 667.8 | 61.8 | 74.6 | 50.2 | 2,035 | 2.0 | 12.0 | 7.3 | 18.5 | 62.6 | 7.3 | 0.4 | 0.08 | 0.8 | 0.7 | 200 |
| Solamar | Zu16 | 1,212.1 | 15.8 | 42.9 | 60.4 | 2,010 | 2.4 | 16.2 | 7.1 | 18.4 | 162.2 | 7.4 | 2.0 | 0.30 | 5.4 | 14.7 | 600 |
| del Diablo | SF1 | 244.2 | 87.6 | 97.8 | 82.4 | 2,137 | 2.1 | 13.6 | 6.6 | 15.1 | 10.2 | 7.2 | 0.5 | 0.12 | 0.2 | 0.9 | 70 |
| Francisco head | SF2 | 390.1 | 99.5 | 99.9 | 100.0 | 2,017 | 2.7 | 22.8 | 6.8 | 13.9 | 6.0 | 7.4 | 0.4 | 0.14 | 0.0 | 1.5 | 90 |
| Navidades | SF3 | 1,032.1 | 83 | 73.6 | 0.0 | 2,033 | 3.1 | 18.3 | 6.6 | 15.2 | 20.6 | 7.9 | 3.1 | 0.36 | 5.2 | 186.5 | 60 |
| Zurita | SF4 | 1128 | 93.2 | 96.5 | 38.3 | 2,078 | 3.4 | 22.6 | 6.6 | 14.1 | 16.0 | 7.7 | 1.0 | 0.14 | 3.2 | 3.9 | 16 |
| Aguirre | SF5 | 302.9 | 85.2 | 90.6 | 82.6 | 2,091 | 2.5 | 13.6 | 7.3 | 15.6 | 31.0 | 8.8 | 0.7 | 0.13 | 0.6 | 3.0 | 20 |
| Pastos | SF6 | 347.1 | 79.5 | 81.3 | 0.0 | 2,000 | 1.9 | 15.5 | 7.4 | 16.1 | 26.8 | 8.2 | 0.7 | 0.16 | 1.4 | 1.4 | 100 |
| San Ramón | SF7 | 463.4 | 97.9 | 99.7 | 77.0 | 1,904 | 2.9 | 20.4 | 6.2 | 15.1 | 9.3 | 7.8 | 0.5 | 0.07 | 0.0 | 0.3 | 50 |
