# Supplementary material for: Deforestation and Benthic Indicators: How Much Vegetation Cover Is Needed to Sustain Healthy Andean Streams?
Source: PLoS One. 2014 Aug 22;9(8):e105869. doi: 10.1371/journal.pone.0105869 (PMC4141824; doi:10.1371/journal.pone.0105869)
Supplement: Table S2 — Stepwise multiple linear regression analysis between macroinvertebrate metrics, land use and stream parameters for 23 Andean streams. Degrees of freedom for all regressions were 1, 20. AIC = Akaike Information Criterion used for selection of predictor variables. DO = Dissolved Oxygen, PO4 3− = Phosphate, TC = Total Coliforms. (DOCX) [file pone.0105869.s002.docx]

Table S2. Stepwise multiple linear regression analysis between macroinvertebrate metrics, land use and stream parameters for 23 Andean streams. Degrees of freedom for all regressions were 1, 20. AIC= Akaike Information Criterion used for selection of predictor variables. DO= Dissolved Oxygen, PO_4_^3¯^= Phosphate, TC= Total Coliforms.

| Dependent | *R^2^* | Overall *p* | AIC | Predictor |
| --- | --- | --- | --- | --- |
| Structural |  |  |  |  |
| Richness | 0.50 | <0.001 | 64.0 | Pasture*  PO_4_^3¯^ |
| Fisher's index | 0.64 | <0.001 | 0.1 | Forest*  DO  Turbidity |
| Evenness | 0.38 | 0.01 | -106.5 | Forest  TC* |
| Compositional |  |  |  |  |
| % Scrappers | 0.28 | 0.01 | 124.4 | Pasture*  TC |
| % Shredders | 0.40 | <0.001 | 28.6 | Forest* |
| Water Quality |  |  |  |  |
| WQI | 0.88 | <0.001 | 62.4 | Pasture*  BOD_5_  Turbidity |
| EPT index | 0.54 | <0.001 | 118.1 | Forest*  PO_4_^3¯^ |
| BMWP/Col | 0.57 | 0.001 | 136.8 | Forest*  DO  Turbidity |
| %5 Dominant taxa | 0.85 | <0.001 | 97.8 | Forest* |
| * Shows the most important predictor based on its contribution to the *R^2^* of the best model. | | | | |
